# Laparoscopic intersphincteric resection with intraoperative radiotherapy using low-energy X-rays for locally advanced ultra-low rectal cancer

**DOI:** 10.1186/s12957-018-1430-6

**Published:** 2018-07-07

**Authors:** Min Wang, Wangsheng Xue, Zeyun Zhao, Yongbo Li, Xinyu Wang, Tao Li, Yongbo Zou, Xiaowei Song, Mingwei Zhang, Tiejun Wang, Jianzheng Yang, Chunyu Wang, Shuang Wang

**Affiliations:** Jilin University Second Hospital, Changchun, Jilin Province China

**Keywords:** Lap ISR, IORT, Low-energy X-rays, Intrabeam X-rays radiotherapy system, Anus preservation

## Abstract

**Background:**

In order to overcome the shortcomings of laparoscopic intersphincteric resection (Lap ISR), an alternative method of delivering intraoperative radiotherapy by Intrabeam X-rays radiotherapy system (XRS) is proposed in this paper. Intrabeam XRS is a device that uses low-energy X-rays source generated by a mobile controller unit, which is featured in accurate irradiation, reduced complications, and less exposure. The purpose of this study is to discuss the feasibility of Lap ISR with intra-operative radiotherapy using low-energy X-rays for locally advanced ultra-low rectal cancer in Asian woman. This novel proposed method will greatly increase the anus preserving probability and improved the quality of life.

**Methods:**

A 53-year-old woman diagnosed with rectal adenocarcinoma had a strong desire to preserve the anal function and presented at the Jilin University Second Hospital, Jilin, China. The tumor’s size was 4 cm × 3 cm. It was located 2 cm from the anus merge and invaded the levator ani muscle. Preoperative clinical staging was T4N1M0 and could be reached R0 resection. After the consent form was signed by the patient, Lap ISR combined with the applicator put through the anus (natural orifice) to the tumor bed was performed and prophylactic ileostomy synchronized the anastomosis. Patient only received 1-cycle chemotherapy regimen of oxaliplatin with capecitabine postoperatively due to personal reasons. Pre- or postoperative radiotherapy was not given.

**Results:**

After clinical follow-up, until now, there is not any sign of local recurrence. Anus function and short-term complications are acceptable. The short-term effect is satisfying and we look forward to further assess the long-term effect.

**Conclusion:**

Laparoscopic intersphincteric resection with IORT using low-energy X-rays for the patients with late-stage ultra-low rectal cancer could provide an opportunity of preserving the anus function, and it is feasible for the selected patients.

**Trial registration:**

Retrospectively registered; Trial registration: NCT03393234; Registered time: 05 January 2017.

## Background

Traditionally, the gold standard of treating locally advanced rectal cancer is total mesentery excision (TME) combined with complementary chemotherapy and radiotherapy [1, 2]. Recently, rectal cancer surgery has focused on the preservation of the sphincter anatomy and function without compromising oncological results, and Lap ISR has been increasingly being popular on saving sphincter with ultra-low rectal cancer patients whose tumor location is less than 5 cm from the anal verge or less than 3 cm from the dente line.

However, preoperative criteria for Lap ISR are the patients diagnosed with clinical stages T1 and T2 and N0_1 [3], and the ultra-low rectal cancer patients in T3~ 4 stage patients are suggested neoadjuvant radiation or chemo-radiation to make this approach possible with a good oncological outcome. Neoadjuvant radiotherapy can downstage the tumor and increase the resectability of stage III or IV, but also have an effect on levator ani muscles and hinder the anal function to recover after surgery [4]. Also, to prevent the postoperative recurrence, patients mostly apply postoperative adjuvant radiotherapy and the period is relatively long.

Nowadays, intra-operative radiotherapy (IORT) is becoming a hot topic in the relevant treatment aiming to improve the local control. A broader area may be covered by the pre- or postoperative external beam radiation. However, with more minimal toxicity and exposure to surrounding structures, IORT directly delivers the designed radiation dosage to at-risk areas removed tumor.

**Fig. 1:**
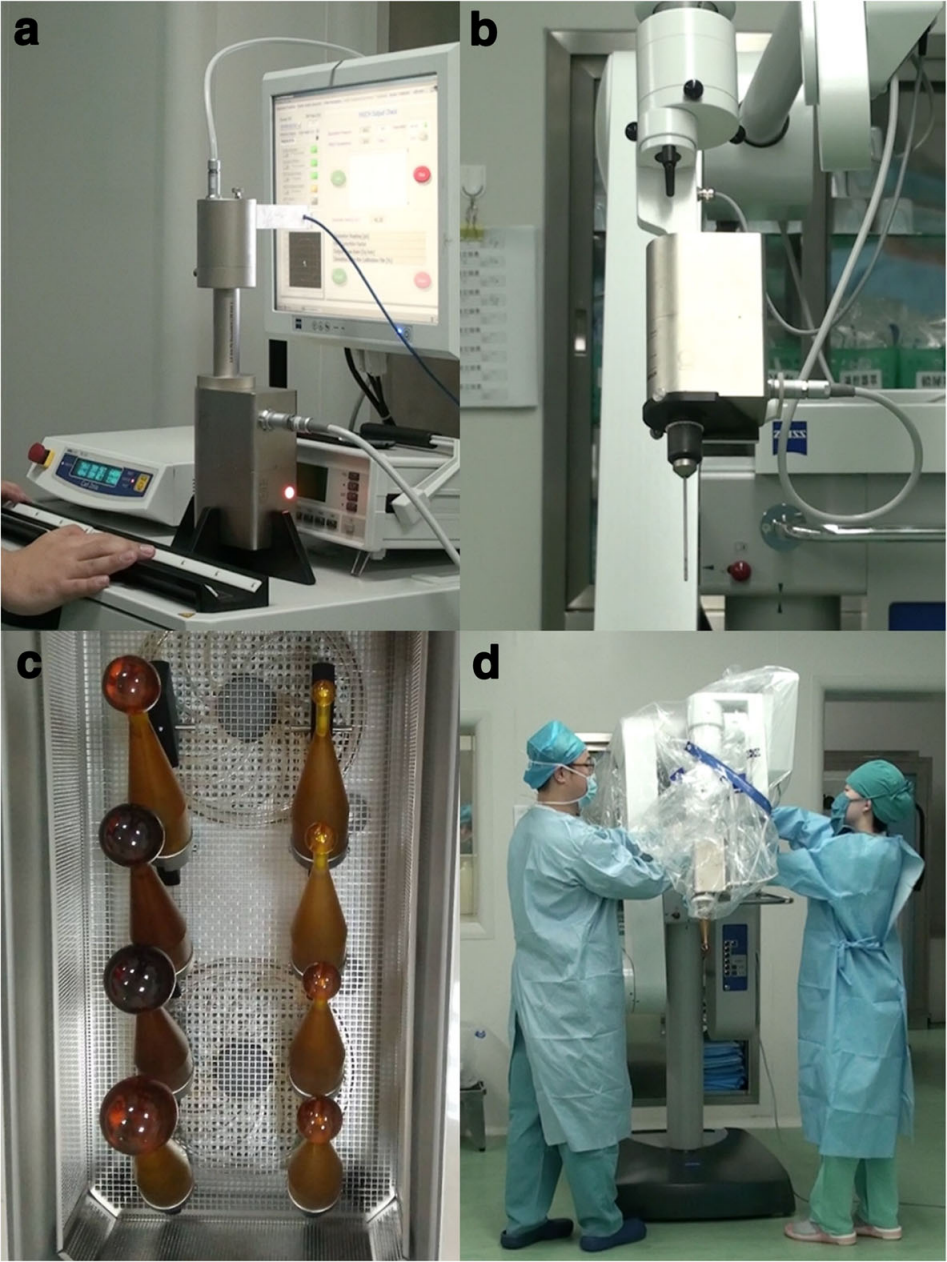
The introduction of Intrabeam System. The Zeiss Intrabeam System (Carl Zeiss Meditec AG, Germany). **a** The console. **b** XRS. **c** Spherical plastic applicators for IORT. **d** The X-rays source (XRS) with a spherical applicator mounted on the floor stand

Several devices for IORT with low-energy X-rays were available. Now, the most common device in use is the Intrabeam X-rays radiotherapy System (XPS) (Carl Zeiss Meditec AG, Germany, Fig. 1). It is feasible and accurate due to special metallic sleeves (Fig. 1b) of the device, which can allow intraoperative radiation by the guiding the drift tube. Therefore, many surgeons attempted to combine the radiotherapy with the surgery. For example, it has been used in kyphoplastic stabilization of vertebral metastases (Kypho- IORT) [5, 6].

Reviewing the literature, few studies involved the Lap ISR with IORT, especially using the Intrabeam X-rays radiotherapy System (XRS). Hence, the combination of the two therapies is a novel, effective, and safe alternative for anus preservation in ultra-low rectal cancer, especially in the late stage.

The purpose of this study was to demonstrate our preliminary experience about the efficacy of the novel method for ultra-low rectal cancer in the selected patients, resulting in good anus preservation outcome and low local recurrence and short-term complications.

## Methods

### Patient

**Fig. 2:**
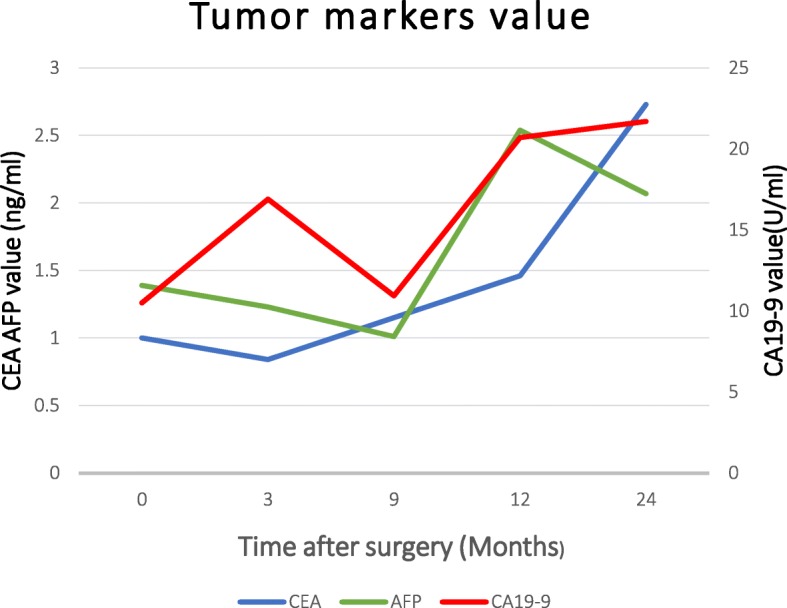
Tumor markers value (CEA: Carcinoembryonic antigen; CA19-9: Carbohydrate antigen19-9)

A 53-year-old woman had symptoms of bloody stool, repeated constipation, defecation habits change, and weight loss for 1 year (Table 1). She was diagnosed with locally advanced rectal cancer in Jilin University Second Hospital, Jilin, China. The tumor was located 2 cm from the anus merge, and its size was 4 cm × 3 cm. The rectum wall was circularly covered around half area, and the pathology was adenocarcinoma by the colonoscopy biopsy. By the pelvic magnetic resonance imaging (MRI), lymph node metastasis was found and there is no distal metastasis detected by the chest X-ray, abdominal computed tomography (CT) scan, and hepatobiliary ultrasound. Particularly, anorectal manometry was required to be as an index measuring its preoperative anal function (Fig. 2).

**Table 1:** Patients demographics and operative outcomes

Characteristics	Patient
Sex	Female
Age	53
Body mass index, kg/m^2^	22.6
Tumor size, cm	4 × 3
Tumor location	Anterior; 2 cm from anus
TNM	T4N1M0
Operating time, min	200
Blood loss, ml	160
Prophylactic stoma	+
Anastomotic leakage	–
Anastomotic stricture	–
IORT dose, Gy	20
IORT time, min	30
Local recurrence	–

*IORT* Intra-operative radiotherapy

The carcinoembryonic antigen (CEA) level of this patient was 1 ng/ml, and CA19–9 level was 10.5 U/ml. She had no family history and other systemic diseases. After she signed the consent form, Lap ISR combined with IORT using low-energy X-rays and prophylactic ileostomy were performed on December 05, 2015, without preoperative chemotherapy or radiotherapy.

### Surgical technique

**Fig. 3:**
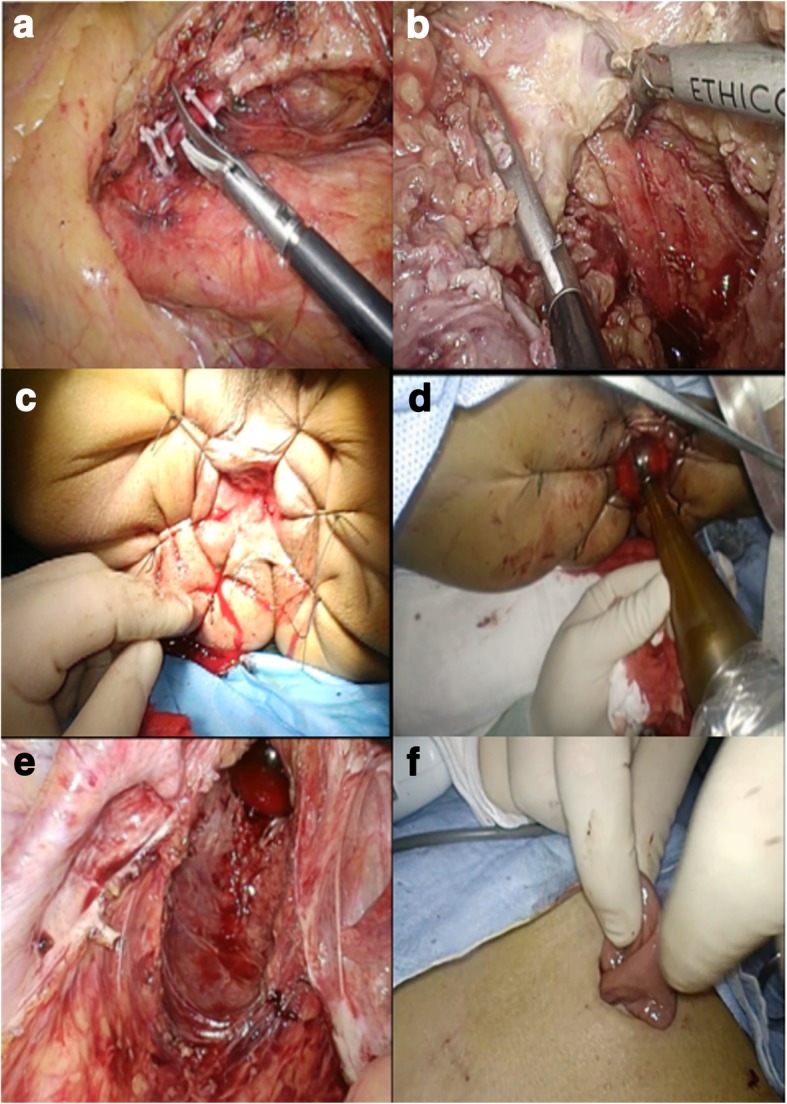
The procedure of surgery. **a** The ligation and excision of the inferior mesenteric artery. **b** Dissection to levator ani muscle. **c** The exposed anal canal by sutures. **d** The applicator was input trans-anally. **e** The applicator put in tumor bed under the surveillance of laparoscopy. **f** The prophylactic ileostomy

When the patient was under general anesthesia in a Lloyd-Davis position, Lap ISR was operated according to the methods described by Schiessel et al. [7]. We performed the operation according to the following procedural steps. Firstly, with the pneumoperitoneum and five ports induced, then laparoscopic exploration was performed. Secondly, we made the patient in the right-head-ventral side position to mobilize the jejunum and the ileum so that we got an optimal surgical view of the left side of the colon. Thirdly, at the origin of the inferior mesenteric artery, ligation was performed and lymphadenectomy around the artery was done at the same time (Fig. 3a). Fourthly, separation and dissection of the left side of the colon were performed by the approach of medial-lateral retroperitoneum. Fifthly, we moved the rectum and excised the mesorectum.

**Fig. 4:**
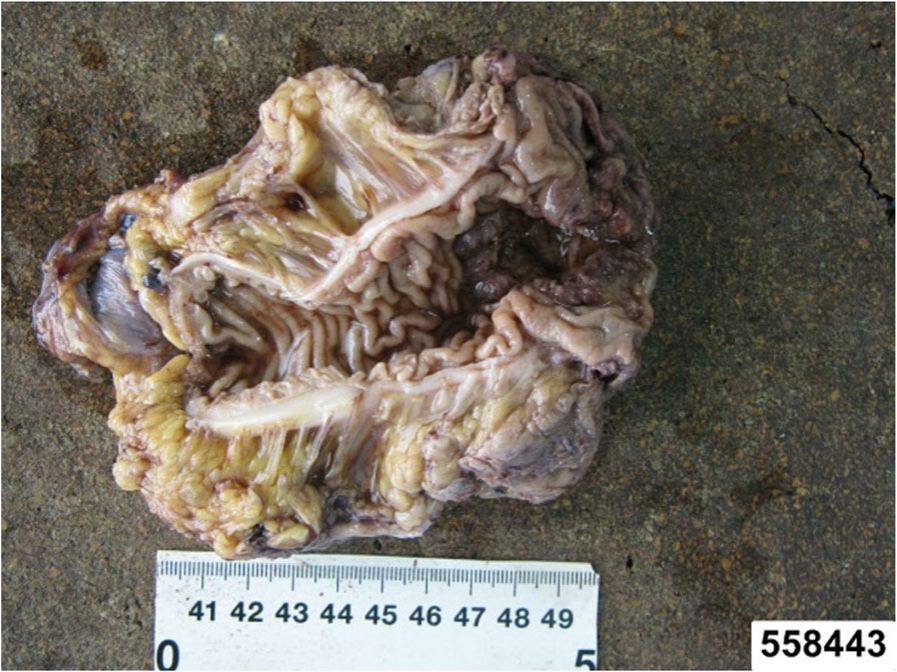
The excision. The excision including the tumor

When the dissection progressed to the endo-pelvic fascia and levator ani muscle (Fig. 3b), the mucosa and involved internal anal sphincter (IAS) were partly circumferentially incised by the trans-anal dissection so that the trans-anal intersphincteric dissection allowed connection with the laparoscopic dissection. At this stage, we cut the proximal rectum (recto-sigmoid section) with a linear stapler (KANGDI, China). At the same time, radiation technician started adjusting the device Intrabeam XRS. Then, the tissue specimen was extracted via the anus (Fig. 4), and the distant incisal edge was sent to intra-operative frozen section examination. The anal canal was exposed by fixing the distal rectum verge to the anus surrounding skin using sutures in six directions (Fig. 3c).

IORT was operated following the anus canal being exposed. Based on the width of the anus canal and the pelvic cavity, applicator at 4 cm in diameter was chosen. Under the surveillance of the laparoscope, surgeon pushed the applicator closely into the tumor bed (specimen location) trans-anally (Fig. 3d) and then surgeon used wet gauze to isolate and protect the surrounding normal structures especially ureter and residual rectum. The applicator surface dose was 20 and 5 Gy in 1 cm in depth as the radiation dosage attenuates evidently with the increase of the distance from the source applicator and the radiation time was 30 min.

Next, anastomosis was made by the circular stapler (Brightness, China) without creating colonic J-pouch following IORT. Finally, to protect the anastomosis, a diverting ileostomy was made routinely (Fig. 3f) and the stoma was closed at the 9th month after surgery. Anal and abdominal drainage tube were set routinely. After surgery, the patient only received once cycle chemotherapy regimen of oxaliplatin combined with capecitabine due to personal reasons and no additional radiotherapy.

### Follow-up

For the first and the 2nd year, the time of postoperative follow-up visit was set at every 3 months and every 6 months, respectively. The patients were followed by a designed standardized questionnaire including results of digital examination, imaging examinations such as computed tomography (CT), and laboratory test including tumor markers (Fig. 2). The evaluation of short-term effect includes complications such as anastomotic leakage, anastomotic stenosis, and urinary dysfunction [8] especially focused on fecal incontinence.

In our study, no matter whether distant metastases occurred. If there was any presence of pelvic, lateral node, or anastomotic recurrences recorded by pathologic or clinical examination, we defined it as local recurrence [9]. A prerequisite for good functional outcome is the continence of the sphincter mechanism. It was evaluated by the pre- and postoperative anorectal manometry which was measured at the postoperative 3rd, 6th, and 12th month. By using this method, we could make a comparison and measured the effect of new treatment generated for sphincter function.

## Results

The whole operative time was about 300 min, including 160 min for Lap ISR, 15 min for putting the applicator, 30 min for radiation time, and 80 min for bowel reconstruction and ileostomy. In 10 days, bowel function recovered and eating occurred. The Foley catheter was removed at the 8th day postoperatively. The anal catheter and abdominal drainage tube were respectively removed at the 14th and the16th day after surgery. Patient was told to dilate the anal canal three to four times slightly by finger a day for early 3 months to prevent the occurrence of the anal and anatomic stenosis. There was no distal margin or circumferential margin (CRM) positive.

**Fig. 5:**
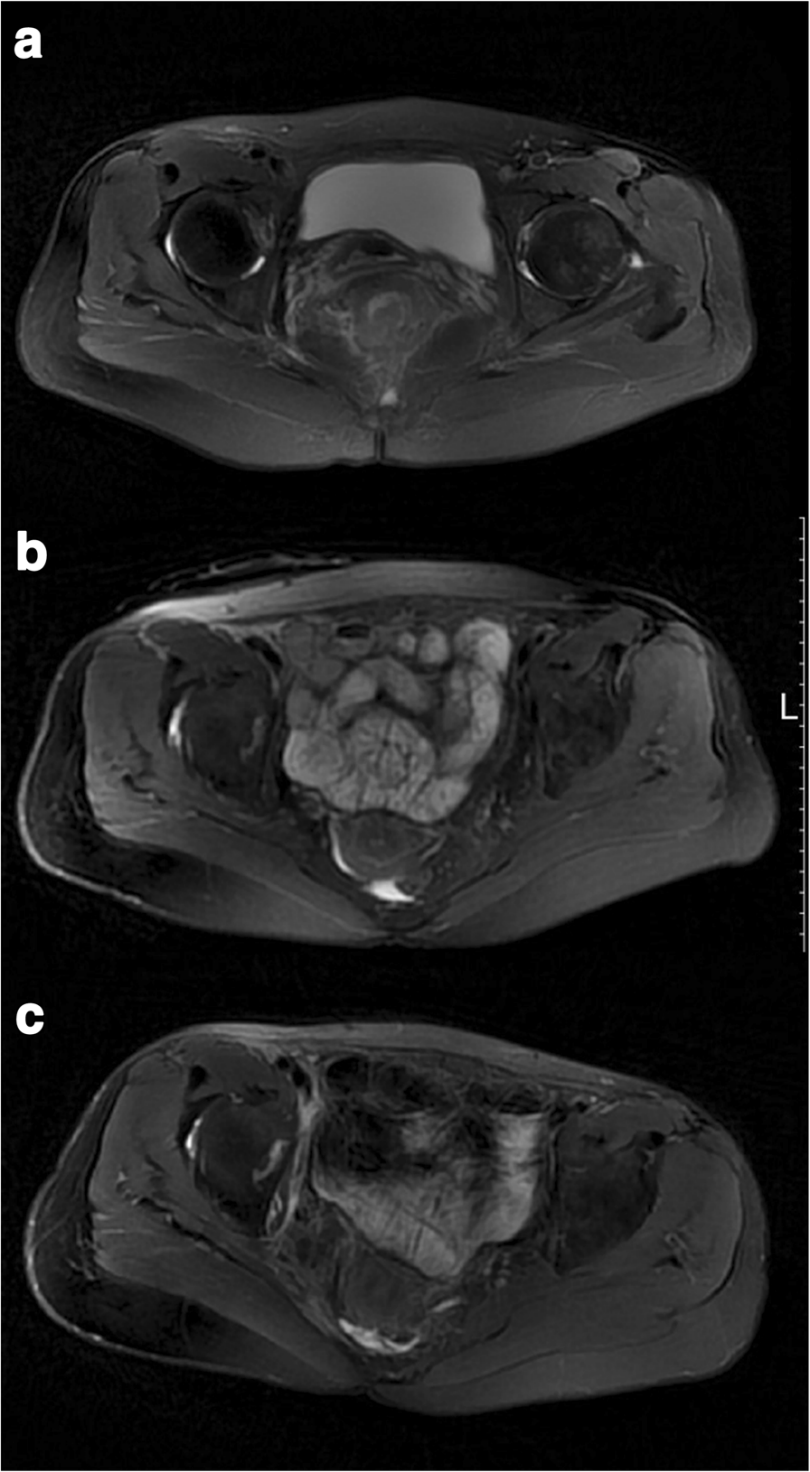
The Pelvic MRI images. **a** Preoperative image. **b** Postoperative 9th month image. **c** Postoperative 24th month image

In the past 2 years, the general condition of the patient was well. There was not any symptom of acute radiation injury (such as in the heart, lung, and circular system). Urinary function was satisfactory as stated in the results of the questionnaire. Anastomotic leakage and stenosis were not found. No local recurrence or distal metastasis occurred (Fig. 5).

Anorectal manometry results (Table 2) illustrated that the postoperative anal function was evidently reduced although no severe complications such as fecal incontinence happened. The data indicated that the anal function was recovering. As related by the patients, symptoms like increased stool frequency and tenesmus were recovering and had a little influence on their life quality. However, the patient was satisfied with the outcome. The long-term effect needs to be further assessed.

**Table 2:** Anorectal manometry results

T		RP (mmHg)	SP (mmHg)
Pre		63	93
Post 3rd M		24	57
Post 6th M		32	63
Post 9th M		45	69
Post 12th M		48	76
Post 18th M		52	82
Post 24th M		54	84

*T* time, *RP* resting pressure, *SP* systolic pressure, *Pre* pre-operation, *Post 3rd M* the third month after operation)

## Discussion

A crucial purpose of Lap ISR is to reserve external sphincter muscle of anus, the levator ani muscle, and part of the internal sphincter muscle of anus for defecation control and to achieve satisfying postoperative life quality. The Intrabeam XRS was first used in the brain tumor irradiation [10]. At present, it is widely used for intraoperative breast irradiation within accelerated partial breast treatment (APBI) [11–13] and also advanced boost treatment [14].

Our study, to our knowledge, is the first to report the use of the Lap ISR combined with IORT with low-energy X-rays for locally advanced ultra-low rectal cancer patients with late stage in laparoscopic colorectal surgery, and we noted several advantages of this treatment as following.

Firstly, with surface applicators [15] and the mechanic arm flexibility at 6° of freedom [16], hence, we could push the spherical plastic applicators into the tumor bed trans-anally, which was input by a natural orifice to avoid any extra abdominal incision for applicator insertion. This advantage reached a satisfied cosmetology, which not only avoided the possibility of wound infection but also made the patient satisfied and acceptable. As well, that greatly shortened the patients’ charge time without the wound infection.

Secondly, Intrabeam X-rays Radiotherapy System(XRS) differs from the Intrabeam with photon radiotherapy system (PRS). Armoogum et al. [17] and Eaton [18] have described many advantages of the Intrabeam XRS. By a miniature 50 kV X-rays generator, it accelerates electrons beam to hit the thin hemispherical gold target from the electron gun down a thin (3.2-mm diameter) drift tube (Fig. 1d). Advantages of this system include its isotropic distribution in the spherical applicator and its higher dose rate of about 10Gy/min for application [16]. That shortens treatment time and could be adjusted to inhibit the proliferation and metastasis of the residual tumor cells. In addition, with the increase distance from the applicator, the dosage attenuated quickly from the applicator surface to deeper normal tissues [16]. That could contribute to better local control and prevent surrounding structures such as the ureter and the ureter dose measurement is on work.

Thirdly, patients were suggested to be performed from 10 to 12 weeks after the end of neoadjuvant treatment [19] and received neoadjuvant radiotherapy total 50 Gy (2 Gy × 25 sessions) before Lap ISR or received postoperative radiotherapy. Compared with that, intraoperative radiotherapy time was greatly shorter than that of the method in Civello et al. [20], and thus, the whole treatment time was also shorter than that. In our study, the effective 20 Gy dosage could be given singly by IORT. The patient whom preoperatively was assessed with R0 resection and received IORT with low-energy X-rays did not need to receive the radiotherapy pre- or postoperatively, which had great advantages on decreasing the visits of hospital and increasing the acceptance of patients.

Fourthly, owing to the multifunctional integrated operation room, multi-department collaboration, and the mobility of Intrabeam XPS, patient avoided transportation and great risks to lose heart beat and get infected, etc. [21, 22]. Also, that made the operation time and stuff energy saved. Meanwhile, there was no extra incision but for the prophylactic ileostomy, which would be closed and bring good appearance and fast recovery and alleviated the postoperative pain as well.

In the past 2 years, we have operated five cases by this novel method. Due to the observed limited time, this study just introduced one case. We hope that more evidences would come to support it when more cases are studied. However, the advantages like the exclusion of non-affected structures from the radiation area, the good outcome of anus preservation, and higher dose homogeneity were proved. Lap ISR with IORT using low-energy X-rays is promising and has a great possibility to be an alternative to the treatment of locally advanced rectal cancer. We believe that more and more surgeons will begin to try this innovative method.

## Conclusion

The innovative treatment modality of the laparoscopic intersphincteric resection with IORT using low-energy X-rays could provide the opportunity of preserving the anus function, and it is feasible for the selected patients. Furthermore, we hypothesized that this method could potentially improve the indications of the Lap ISR and benefit the patients with locally advanced ultra-low rectal cancer. It also should be considered as an option when operating on these challenging patients.

## Abbreviations

CA19-9: Carbohydrate antigen19-9
CEA: Carcinoembryonic antigen
CRM: Circumferential margin
IAS: Internal anal sphincter
IORT: Intraoperative Radiotherapy
Lap ISR: Laparoscopic intersphincteric resection
Post 3th M: The third month after operation
Pre: Pre-operation
RP: Resting Pressure
SP: Systolic Pressure
T: Time
XRS: X-rays radiotherapy System
